# The Rising Dominance of *Shigella sonnei*: An Intercontinental Shift in the Etiology of Bacillary Dysentery

**DOI:** 10.1371/journal.pntd.0003708

**Published:** 2015-06-11

**Authors:** Corinne N. Thompson, Pham Thanh Duy, Stephen Baker

**Affiliations:** 1 The Hospital for Tropical Diseases, Wellcome Trust Major Overseas Programme, Oxford University Clinical Research Unit, Ho Chi Minh City, Vietnam; 2 Centre for Tropical Medicine, Nuffield Department of Clinical Medicine, Oxford University, Oxford, United Kingdom; 3 The London School of Hygiene and Tropical Medicine, London, United Kingdom; University of Queensland, AUSTRALIA

## Abstract

Shigellosis is the major global cause of dysentery. *Shigella sonnei*, which has historically been more commonly isolated in developed countries, is undergoing an unprecedented expansion across industrializing regions in Asia, Latin America, and the Middle East. The precise reasons underpinning the epidemiological distribution of the various *Shigella* species and this global surge in *S*. *sonnei* are unclear but may be due to three major environmental pressures. First, natural passive immunization with the bacterium *Plesiomonas shigelloides* is hypothesized to protect populations with poor water supplies against *S*. *sonnei*. Improving the quality of drinking water supplies would, therefore, result in a reduction in *P*. *shigelloides* exposure and a subsequent reduction in environmental immunization against *S*. *sonnei*. Secondly, the ubiquitous amoeba species *Acanthamoeba castellanii* has been shown to phagocytize *S*. *sonnei* efficiently and symbiotically, thus allowing the bacteria access to a protected niche in which to withstand chlorination and other harsh environmental conditions in temperate countries. Finally, *S*. *sonnei* has emerged from Europe and begun to spread globally only relatively recently. A strong selective pressure from localized antimicrobial use additionally appears to have had a dramatic impact on the evolution of the *S*. *sonnei* population. We hypothesize that *S*. *sonnei*, which exhibits an exceptional ability to acquire antimicrobial resistance genes from commensal and pathogenic bacteria, has a competitive advantage over *S*. *flexneri*, particularly in areas with poorly regulated antimicrobial use. Continuing improvement in the quality of global drinking water supplies alongside the rapid development of antimicrobial resistance predicts the burden and international distribution of *S*. *sonnei* will only continue to grow. An effective vaccine against *S*. *sonnei* is overdue and may become one of our only weapons against this increasingly dominant and problematic gastrointestinal pathogen.

## Introduction

Shigellosis, caused by members of the bacterial genus *Shigella*, is a severe and occasionally life-threatening diarrheal infection. Worldwide, *Shigella* spp. are the most common cause of acute, bloody diarrhea (dysentery) and are responsible for a significant proportion of the burden of morbidity and mortality associated with diarrheal disease [1,2]. In Asia alone, it is estimated that there are 125 million infections and 14,000 deaths due to shigellosis annually [3]. As a result of the considerable global burden, low infectious dose [4], clinical severity, and frequent reports of emerging antimicrobial resistance against first- and, more recently, second-line therapies [5,6], a vaccine against *Shigella* infections is a growing necessity. Yet, more than a century after the discovery of the agent of bacillary dysentery, there is still neither a licensed vaccine nor agreement on the precise mechanisms that induce *Shigella* immunity [7]. Vaccine development is further complicated by the probable need for a multivalent combination of O polysaccharide antigens to protect against a variety of heterogeneously distributed serotypes [8].

The genus *Shigella* incorporates four species. *Shigella dysenteriae* was historically responsible for large epidemics [9] yet is now rarely identified [8]. Similarly, *S*. *boydii* is also infrequently isolated. *S*. *flexneri*, however, is common globally and traditionally isolated most frequently in resource-poor countries [10]. *S*. *flexneri* has 15 different serotypes distributed heterogeneously across different regions, with predominant serotypes including *S*. *flexneri* 2a, 3a, and 6 [8,11]. Finally, *S*. *sonnei* is also prevalent globally, although traditionally most commonly detected in high-income regions [10,12]. *S*. *sonnei* has only one serotype.

## *S*. *sonnei*: An Emergent Pathogen

**Fig 1:**
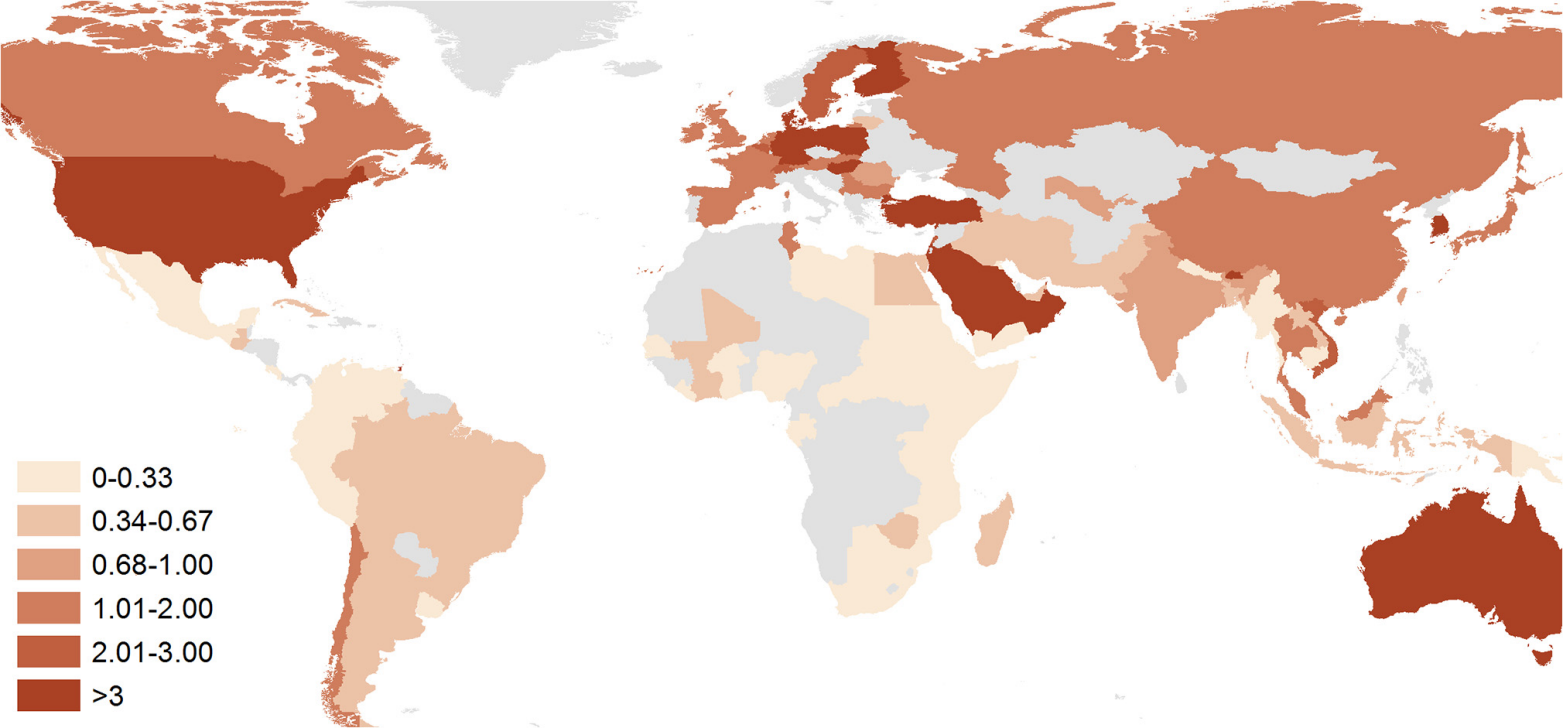
The ratio of *S*. *sonnei* to *S*. *flexneri* isolated from 100 countries, 1990–2014. The darker the color, the higher the proportion of *S*. *sonnei* isolated from each country; the lighter the color, proportionally higher the proportion of *S*. *flexneri* isolated. Countries colored grey indicate no data on species were identified. To generate this map, we performed an extensive literature review in PubMed using the term “*Shigella*” followed by the name of 178 countries. The most recent publication that included species information that was nonoutbreak and nontravel associated was included as representative of each country. If country data were pre-1990 or were not available on PubMed, the Gideon Infectious Disease encyclopedia as well as national reference laboratory data were referenced where possible. References are listed by country in the supplementary material (S1 Table).

Reasons behind the conventional dominance of *S*. *sonnei* in industrialized countries remain unclear [13]. However, an increasing proportion of shigellosis due to *S*. *sonnei* generally correlates with improving economic prosperity [12], which in the context of many rapidly developing countries, has led to a proportional decrease in *S*. *flexneri* and the simultaneous emergence of *S*. *sonnei* [14]. This shift toward *S*. *sonnei* has been documented in many regions in Asia, Latin America, and the Middle East (Fig 1) [15–20], with proven explanations behind such an epidemiological phenomenon lacking. This review aims to summarize the existing evidence as to why *S*. *sonnei* may predominate in high-income countries and why it is now emerging in regions traditionally dominated by *S*. *flexneri* and explores the implications of the growing threat of this increasingly antimicrobial-resistant pathogen for public health globally.

## *Plesiomonas shigelloides*: Passive Environmental Immunization?

One of the principal theories regarding the lack of *S*. *sonnei* in industrializing areas focuses on the Gram-negative bacteria *P*. *shigelloides*, which like *Shigella* falls within the large eubacterial family of the Enterobacteriaece. *P*. *shigelloides* and *S*. *sonnei* share an identical lipopolysaccharide (LPS) O-side chain (confirmed by nuclear magnetic resonance [NMR] and mass spectrometry) that is thought to be the major surface antigen targeted by the adaptive immune system during *Shigella* infection [21,22]. These surface antigens are cross-reactive, and vaccines prepared from O-antigen derived from *P*. *shigelloides* have been shown to be reasonably effective in preventing infection with *S*. *sonnei* in humans [23,24]. The O-antigen gene cluster is located on the *S*. *sonnei* invasion plasmid and is essential for penetration of host epithelial cells [25]. Evidence suggests not only that *S*. *sonnei* acquired the O-antigen gene cluster from *P*. *shigelloides* but also that this acquisition was the defining event in the emergence of *S*. *sonnei* [26].

Due to the cross-reactive nature of the *S*. *sonnei/P*. *shigelloides* O-antigens, Sack and colleagues suggested that exposure to *P*. *shigelloides* serotype O17 leads to protection against infection with *S*. *sonnei* [27]. In areas with poor-quality water supplies, the authors postulated that exposure to *P*. *shigelloides* occurs frequently and thus disease due to *S*. *sonnei* is rare, as the population is effectively naturally immunized [27]. Although *P*. *shigelloides* is found in water and environmental samples in both industrialized and industrializing countries [28,29], water treatment practices are likely to prevent frequent exposure in regions with adequate sanitation. Highlighting this, outbreaks of diarrheal disease thought to be due to *P*. *shigelloides* occurred after a lapse in water chlorination in Japan in the mid-1970s [30]. Although the serotypic distribution of *P*. *shigelloides* has not been well described [31], serotype O17 has been reported in both water and stool samples from patients admitted for diarrhea in industrializing regions [32,33], lending credence to the hypothesis of water-driven immunization at the population level.

The phenomenon of passive immunization in low-income countries would explain, at least in part, why *S*. *sonnei* is proportionally more commonly isolated in industrialized countries. Accordingly, an increase in the proportion of *Shigella* episodes due to *S*. *sonnei* would occur concurrently with economic development and improved water supplies [27]. Ram and colleagues confirmed this economic trend by identifying a strong positive correlation between country-level GDP and proportion of isolated *Shigella* due to *S*. *sonnei* from 56 studies conducted from 1984–2005 (R = 0.55, *p* < 0.0001) [12]. Therefore, the combination of improving economic outlook and fulfillment of the Millennium Development Goals (MDGs) will lead to an improvement of drinking water supply, a drop in population-level cross protection against *S*. *sonnei*, and potentially to a global increase in *S*. *sonnei* infections in heavily populated regions currently undergoing such transitions [34].

## *Acanthamoeba*: An Environmental Host?

*Acanthamoeba* is the most common amoeba found globally, with a wide distribution in both aquatic and nonaquatic environments [35]. Amoeba such as *Acanthamoeba* are known to act as environmental hosts for a variety of intracellular pathogens including *Helicobacter pylori*, *Vibrio cholerae*, and also various *Shigella* spp. [36–38]. The uptake of bacteria into amoebic cysts allows the bacteria to persist in adverse environmental conditions, including desiccation, starvation, and a variety of chemical and physical agents [39]. *Acanthamoeba* cysts, which form when triggered by nutritional or osmotic stress, are particularly resistant to chlorine treatment [40]. Found commonly through environmental sampling [41], *Acanthamoeba* has been identified in public water supplies in developed countries with appropriate chlorination levels [42] and in hospital water supplies [43] and can also be isolated from drinking water supplies in industrializing regions [44].

Recent evidence confirms that *S*. *sonnei*, *S*. *dysenteriae*, and *S*. *flexneri* can be taken up by the species *Acanthamoeba castellanii* when grown under laboratory conditions [36,45,46]. Once phagocytized, *Shigella* spp. are localized in *A*. *castellanii* vacuoles and eventually in the cysts [36,45] and can survive for over three weeks [45]. Notable differences in symbiotic growth were recorded with respect to amoebic uptake between *Shigella* species. *S*. *sonnei* has been shown to be efficiently taken up and maintained by *A*. *castellanii* at temperatures between 18–30°C [36,46]; indeed, growth of *S*. *sonnei* in the presence of *Acanthamoeba* was found to exceed that of *S*. *sonnei* cultured alone [46]. *S*. *flexneri*, however, was found to significantly inhibit *A*. *castellanii* growth in the laboratory at 30°C [46]. Inhibition and killing of *A*. *castellanii* by *S*. *flexneri* is due to activation of invasion genes, which may induce apoptosis through the secretion of effector proteins into the host cell via the type three secretion system [47,48].

The growth and survival rates of *Shigella* in the cytoplasm of the amoeba resemble their pattern of growth and survival in mammalian macrophages [45,49]. In fact, it has been suggested that growth in the amoebic intracellular niche may have influenced the ability of *Shigella* to survive in the mammalian phagocytic cell environment [50]. Additionally, as free-living amoeba feed on bacteria, fungi, and algae, lateral gene transfer within the amoeba phagolysosome may have facilitated genetic adaptations that allow for the expression of pathogenic or symbiotic phenotypes based on impact on the host cell [51,52]. It has been further suggested that amoebae themselves represent an important genetic reservoir for internalized microbes [51]. For example, there has been an observed increase in resistance to various antimicrobials and biocides in *Legionella pneumophila* grown within free-living amoeba [53], which may be due to selection within the amoeba itself [51].

All aspects considered, available evidence suggests that *A*. *castellanii* may contribute to transmission of *S*. *sonnei* in temperate regions by phagocytizing *S*. *sonnei*, thus allowing the bacteria to circumvent the effects of chlorination and good sanitation [54]. As *S*. *flexneri* has been shown to inhibit growth of *A*. *castellani* [46], the amoeba may not be a viable reservoir for *S*. *flexneri* in either developed or industrializing countries. Protozoa appear to play an important role in the transition of bacteria from the environment to mammals and as such may be the source of emerging pathogenic bacteria [50], and may play an increasing role in the epidemiology of *S*. *sonnei* in industrializing regions as the prevalence increases.

## Antimicrobial Resistance: A New Defense Strategy?

Phylogeographical analyses of a large number of *S*. *sonnei* isolates spanning several continents and several decades in a publication by Holt et al. demonstrated that all contemporary *S*. *sonnei* infections are due to a small number of clones that dispersed globally from Europe within the last 500 years [34]. Four distinct lineages of *S*. *sonnei* were identified, with lineage III the most prevalent globally, becoming dominant in Asia, Africa, and South America [34]. *S*. *sonnei* belonging to lineage III are characterized by the presence of distinct class II integron (In*2*), which confers resistance to trimethoprim, streptothricin, and streptomycin [55]. Many lineage III isolates were also found to harbor a genetic locus on a small plasmid conferring resistance to a variety of additional antimicrobials including tetracycline and sulphonamides. Holt and colleagues indicated that In*2* was likely acquired during the mid-20th century, after which the clone spread internationally, undergoing contemporary global dispersal and localized clonal expansions [34].

This localized microevolution of *S*. *sonnei* appears to be largely driven by selection pressure induced by antimicrobials [56]. The determinants for antimicrobial resistance in *Shigella* are generally located on mobile genetic elements such as plasmids, transposons, and integrons [55]. Horizontal gene transfer (HGT) of such elements is now recognized to be an important driver of bacterial evolution [57,58]. One study, for example, estimated 18% of the 4,288 genes of *Escherichia coli* strain MG1655 were acquired laterally since the species diverged from the *Salmonella* lineage 100 million years (Myr) ago, a rate of 16 kb/Myr/lineage [59]. Transfer of mobile genetic elements between members of the Enterobacteriaceae is known to be responsible for the dissemination of antimicrobial resistance genes and the emergence of a variety of multidrug-resistant (MDR) Gram-negative bacteria globally [55,60,61].

*S*. *sonnei* can acquire advantageous chromosomal and plasmid-mediated resistance genes through HGT from both commensal and pathogenic bacteria circulating locally, enhancing its ability to establish infection, prolonging shedding, and, presumably, outcompeting antimicrobial-susceptible bacteria [56]. A study of >250 *S*. *sonnei* isolates collected over 15 years in Vietnam documented the rapid emergence and dominance of successful clones of *S*. *sonnei* after the acquisition and fixation of plasmids conferring colicin production/immunity and resistance to third-generation cephalosporins in two separate genetic bottleneck events [56]. In areas with unregulated antimicrobial use, *S*. *sonnei* may have abundant opportunity to acquire locally derived resistance genes [62]. In countries with restricted antimicrobial usage, for example, *S*. *sonnei* are generally more susceptible to quinolones [5], presumably because of lower selective pressure combined with reduced availability of resistance genes in the circulating accessory gene pool. Such a phenomenon is thought to be leading to increasingly successful clones in areas of unregulated antimicrobial use and could lead to a rapidly growing and increasingly challenging public health problem in many industrializing areas [56].

In 2005, the WHO published guidelines recommending ciprofloxacin to be used as the first-line treatment for dysentery [63]. The late 2000s saw the first documented resistance in *S*. *sonnei* against fluoroquinolones [5]. Phylogeographical data from Holt and colleagues indicate marked differences in the global prevalence of *gyrA* (DNA gyrase) mutations, which confer resistance to quinolones and reduced susceptibility to fluoroquinolones. Strong selection for quinolone resistance was identified, as the facilitating mutations have occurred independently on multiple occasions in several different lineages and genetic locations [34]. Unless the use of fluoroquinolones becomes regulated in areas of current unrestricted use, ciprofloxacin will likely become ineffective for treating *Shigella* infections in the near future [5]. Yet pivmecillinam, ceftriaxone, or azithromycin may be effective alternatives [64,65], depending on local resistance patterns.

## Does *Shigella sonnei* Have a Competitive Advantage?

**Fig 2:**
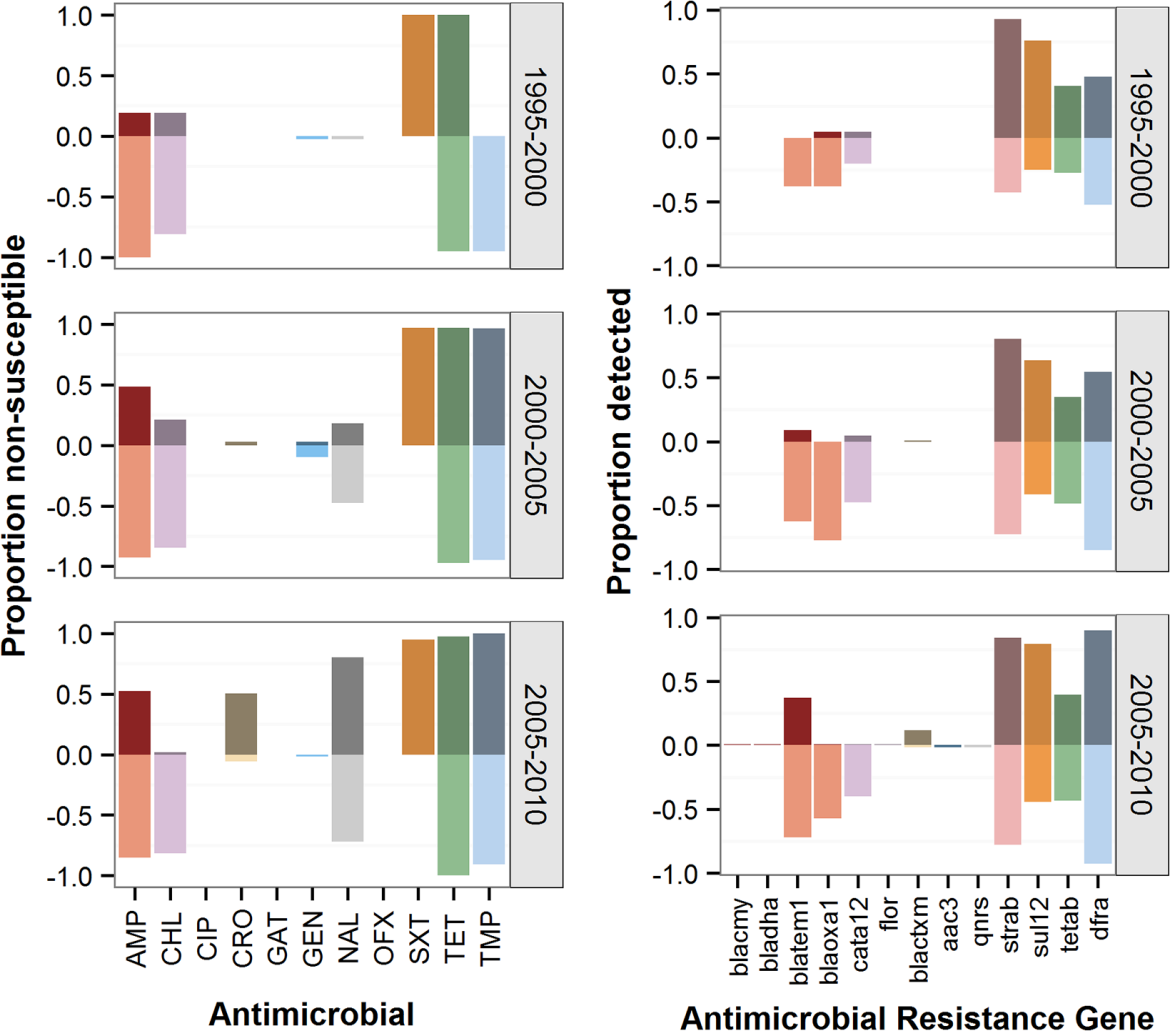
Antimicrobial resistance and presence of resistance-conferring genes in *S*. *sonnei* and *S*. *flexneri*. Plots on the left show proportion of antimicrobial resistance determined by minimum inhibitory concentration (MIC) amongst isolates collected from Vietnam over a 15-year period (*n* = 231 for *S*. *sonnei* and 136 for *S*. *flexneri*) [34,56]. *S*. *sonnei* are shown by darker colors on the top of each graph, and *S*. *flexneri* is shown by lighter colors at the bottom. Plots on the right show the proportion of *S*. *sonnei* (dark color, top of each graph) and *S*. *flexneri* (light color, bottom of each graph) found to have varying resistance genes present on either plasmids or the chromosome. See Supporting Information (SI) S1 Text for a description of procedures. The color of the gene corresponds with the color of the antimicrobial to which it confers resistance on the left. AMP: ampicillin; CHL: chloramphenicol; CIP: ciprofloxacin; CRO: ceftriaxone; GAT: gatifloxacin; GEN: gentamicin; NAL: nalidixic acid; OFX: ofloxacin; SXT: cotrimoxazole; TET: tetracycline; TMP: trimethoprim.

Taken together, evidence suggests that the global burden of *S*. *sonnei* may be growing compared to that of *S*. *flexneri*. This phenomenon may not only be due to the global improvements in water quality and an ability of *S*. *sonnei* to grow successfully within *Acanthamoeba* but may also be due to a potential, but as yet unproven, ability to acquire and/or maintain a wider array of antimicrobial resistance genes. Indeed, it has been speculated that the plasmid composition and resistance profiles may differ between the *Shigella* species isolated from contemporaneous patient populations in the same locations (Fig 2) [15,66–71]. Although *S*. *sonnei* can acquire extended-spectrum beta-lactamase (ESBL)-mediated resistance from other Enterobacteriaceae, particularly *E*. *coli* and *Klebsiella* spp. [72], it is not currently known whether *S*. *flexneri* and *S*. *sonnei* acquire resistance genes from each other. Toro et al. suggested that there is a greater restriction barrier for conjugal plasmids between *S*. *sonnei* and *S*. *flexneri* than between other Gram-negative donors and recipients [67]. A differential ability to acquire and/or maintain plasmids between *S*. *flexneri* and *S*. *sonnei* from other bacterial donors may also explain discrepant resistance profiles between contemporaneous species, although such a phenomenon has yet to be explicitly investigated.

Levels of inflammation in the gut during infection could explain differences in the ability to acquire mobile genetic elements of resistance between the species. Stecher et al. demonstrated that inflammatory responses in the gut during infection may facilitate conjugative transfer and reassortment of plasmid-encoding genes between pathogens and commensal organisms [73]. Although it was shown recently that *S*. *flexneri* is able to modify its LPS structure to dampen the inflammatory innate immune response to allow it to successfully evade detection in the initial phases of infection [74], it is not yet known whether this occurs during *S*. *sonnei* infection. Investigations into differential inflammatory response between the *Shigella* species during infection and their relationship to HGT within the human gastrointestinal tract are warranted.

Finally, *S*. *sonnei* has been shown to be genetically more similar to its ancestor *E*. *coli* than to other *Shigella* species [75,76]. Gene retention from *E*. *coli* potentially imbues *S*. *sonnei* with a higher likelihood for survival in the environment or an environmental adaptive host [75], such as *Acanthamoeba*. *S*. *flexneri*, however, loses genes faster than any other *Shigella* species and is the most genetically distant of the *Shigellae* from *E*. *coli* [76]. Hershberg and colleagues suggest a potential “point of no return” for *Shigella* in that once it undergoes enough purifying selection, it cannot regain enough of its lost functionality to escape niche limitation [76]. Does enhanced capacity for genomic plasticity explain the hypothesized increased ability of *S*. *sonnei* to acquire or maintain plasmids from other bacterial donors [77]? Experimental evidence of differences in gene acquisition and retention between the two species is needed.

## Next Steps

We predict that the combination of improving water supplies and rapid acquisition and maintenance of mobile elements conferring advantageous resistance genes is accelerating a *Shigella* species shift toward *S*. *sonnei* dominance, which traditionally has been shown to occur over a period of decades in individual countries (Table 1, S1 Table) [78,79]. To counter this rapid species replacement, many questions regarding the epidemiology of *S*. *sonnei* and, crucially, vaccine development need to be addressed. Identifying prominent transmission routes of *S*. *sonnei* and *S*. *flexneri* in resource-poor countries should remain a primary goal. Indeed, experiments to determine the relative fitness of each species in varying environmental conditions and investigating antimicrobial fitness [80] would provide information on potential niche preferences and add insight into which accessory gene pools each species samples from. Finally, longitudinal monitoring of water supplies for the presence of both *P*. *shigelloides* and *A*. *castellanii* would help to verify the hypotheses presented in this review in regard to both a reduction of population immunity against the *S*. *sonnei* O-antigen as well as an environmental amoeba niche for these bacteria.

Furthermore, research on the genetic structure of global *S*. *flexneri* populations is warranted in order to help further understand the global species shift and to explore the global and localized microevolution of this pathogen over time. Such analyses would help to predict the role of *S*. *flexneri* in the context of improving sanitation and growing prevalence of *S*. *sonnei* worldwide. Finally, although plagued with many setbacks [11], the development of a sufficiently safe and effective *S*. *sonnei* vaccine may be feasible in the coming decade. However, in order to carry out properly designed vaccine trials in the future, outstanding questions regarding correlates of immunity, incidence in the community, seroconversion rates, and the role of maternal antibody in the first years of life will need to be answered.

**Table 1:** Summary of factors behind the traditional and current epidemiological distribution of *S*. *sonnei* and *S*. *flexneri*.

Factor	Region			
		Industrializing		Industrialized
Explanation of traditional geographical distribution	1.	*S*. *sonnei* is not present because of population immunity due to cross protection from exposure to *P*. *shigelloides* found in contaminated water supplies [26]	1.	*S*. *sonnei* is present because of a lack of cross protection from exposure to *P*. *shigelloides* due to clean water supplies
	2.	*S*. *flexneri* is not able to grow within the common amoeba *A*. *castellanii* [46]	2.	*S*. *sonnei* is symbiotically phagocytosed by *A*. *castellanii* and can withstand chlorination and other harsh environmental conditions [36]
Why is the burden of *S*. *sonnei* growing?	1.	Improving water supplies may lead to a decrease in the prevalence of *P*. *shigelloides*, resulting in lack of cross protection against *S*. *sonnei* [27]		
	2.	Expansion of *S*. *sonnei* from Europe in the last 500 years and subsequent microevolution due largely to local antimicrobial use [34,56]		
	3.	Proposed competitive advantage of *S*. *sonnei* against *S*. *flexneri* due to an enhanced ability to acquire and maintain mobile resistance genes from other bacterial species		

In conclusion, *S*. *sonnei* represents an emerging threat to public health globally. With continuing efforts for improvements in water and sanitation worldwide, population-level immunization against *S*. *sonnei* due to exposure to *P*. *shigelloides* is declining. Additionally, environmental hosts such as *A*. *castellani* represent an important yet potentially overlooked reservoir of *S*. *sonnei* and may explain in part the persistence of *S*. *sonnei* in regions with a reasonably good standard of sanitation. Finally, the incredible ability of *S*. *sonnei* to acquire resistance to a variety of widely used antimicrobials may endow the pathogen with a competitive advantage over sensitive bacterial competitors and predicts its emergence in areas with unregulated antimicrobial use. Combined, this evidence suggests alarming increases in global prevalence of *S*. *sonnei* and unprecedented levels of resistance, demanding a vaccine in the near future that can be administered to the most vulnerable populations, particularly young children in rapidly industrializing countries.

## Boxes

**Box 1. Key Learning Points**

1. Traditionally, the various species of the bacterial genus *Shigella* have a distinct geographical distribution. *S*. *sonnei* is most commonly isolated in industrialized countries, whereas *S*. *flexneri* is more commonly isolated in industrializing regions. However, *S*. *sonnei* is now becoming recognized as a common enteric pathogen in many industrializing regions. The exact mechanisms catalyzing this shift in the epidemiological distribution are unclear.
2. Improving the quality of drinking water supplies in industrializing regions is likely to reduce cross protection against *S*. *sonnei* derived from the bacterium *P*. *shigelloides*, which is commonly found in contaminated water.
3. *S*. *sonnei* may be efficiently phagocytized by the ubiquitous amoeba species *A*. *castellani*, thereby providing it with a reservoir in which to withstand chlorination and other harsh environmental conditions.
4. In comparison to *S*. *flexneri*, *S*. *sonnei* has a greater ability to develop resistance to broad-spectrum antimicrobials. We suggest that *S*. *sonnei* is more likely to accept and maintain horizontally transferred DNA, which gives it a competitive advantage against *S*. *flexneri*, particularly in areas with unregulated antimicrobial use.
5. With ongoing improvements in the international quality of water supplies and rapid development of antimicrobial resistance, the burden of *S*. *sonnei* is likely to grow substantially. A vaccine against *S*. *sonnei* is increasingly necessary.

**Box 2. Top Five Papers**

1. Sack D, Hoque A, Huq A, Etheridge M (1994) Is Protection against Shigellosis Induced by Natural Infection with *Plesiomonas shigelloides*? Lancet 343: 1413–1415.
2. Saeed A, Johansson D, Sandström G, Abd H (2012) Temperature Depended Role of *Shigella flexneri* Invasion Plasmid on the Interaction with *Acanthamoeba castellanii*. Int J Microbiol 2012: 917031.
3. Holt KE, Baker S, Weill F-X, Holmes EC, Kitchen A, et al. (2012) *Shigella sonnei* Genome Sequencing and Phylogenetic Analysis Indicate Recent Global Dissemination from Europe. Nat Genet 44: 1056–1059.
4. Holt KE, Thieu Nga TV, Thanh DP, Vinh H, Kim DW, et al. (2013) Tracking the Establishment of Local Endemic Populations of an Emergent Enteric Pathogen. Proc Natl Acad Sci 110: 17522–17527.
5. Levine MM, Kotloff KL, Barry EM, Pasetti MF, Sztein MB (2007) Clinical Trials of *Shigella* Vaccines: Two Steps Forward and One Step Back on a Long, Hard Road. Nat Rev Microbiol 5: 540–553.

## Supporting Information

S1 Table Country-specific references for Fig 1 showing the ratio of *S*. *sonnei* to *S*. *flexneri* isolated from 100 countries, 1990–2014. (DOCX)

S1 Text Methods for antimicrobial resistance and gene content data shown in Fig 2. Data are derived from analyses of 367 *Shigella* isolates collected between 1995 and 2010 from Vietnam (136 *S*. *flexneri* and 231 *S*. *sonnei*). Resistance was determined by MIC and gene content analysis from Illumina genome sequencing data.
(DOCX)
