# Supplementary material for: The Rising Dominance of Shigella sonnei: An Intercontinental Shift in the Etiology of Bacillary Dysentery
Source: PLoS Negl Trop Dis. 2015 Jun 11;9(6):e0003708. doi: 10.1371/journal.pntd.0003708 (PMC4466244; doi:10.1371/journal.pntd.0003708)
Supplement: S1 Table — (DOCX) [file pntd.0003708.s001.docx]

**Table S1. Country-specific references for figure 1 showing the ratio of *S. sonnei* to *S. flexneri* isolated from 100 countries, 1990-2014.**

Listed in alphabetical order by country. Missing countries did not have any publically available data >1990.

| **Country** | **Reference** |
| --- | --- |
| Argentina | Rolfo F, Marin GH, Silberman M, Pattin J, Gatti B, et al. (2012) Epidemiological study of shigellosis in an urban area of Argentina. J Infect Dev Ctries 6: 324–328. |
| Australia | Australia’s Notifiable Disease Status, 2009: Annual Report of the National Notifiable Diseases Surveillance System (2009). Canberra, Australia. Available at http://www.health.gov.au/internet/main/publishing.nsf/ Content/cda-pubs-annlrpt-nndssar.htm |
| Austria | Bundesministerium Fur Gesundheit: Mitteilungen fur das offentliche Gesundheitswesen (Public Health Newsletter), Ausgabe 3, Quartal 2014 (2014). Wien, Austria. Available at http://bmg.gv.at/cms/home/attachments  /0/2/1/CH1184/CMS1412325626202/nl_3_quartal_2014_neuerliche_aussendung_23102014.pdf |
| Bahrain | Jamsheer A, Bindayna K, Al-Balooshi N, Botta G (2003) Trend of antibiotic resistance in 1316 *Shigella* strains isolated in Bahrain. Saudi Med J 24: 424–426. |
| Bangladesh | Livio S, Strockbine NA, Panchalingam S, Tennant SM, Barry EM, et al. (2014) *Shigella* isolates from the global enteric multicenter study inform vaccine development. Clin Infect Dis 59: 933–941. |
| Belgium | Vrints M, Mairiaux E, Van Meervenne E, Collard J-M, Bertrand S (2009) Surveillance of antibiotic susceptibility patterns among *Shigella sonnei* strains isolated in Belgium during the 18-year period 1990 to 2007. J Clin Microbiol 47: 1379–1385. |
| Bhutan | Ruekit S, Wangchuk S, Dorji T, Tshering KP, Pootong P, et al. (2014) Molecular characterization and PCR-based replicon typing of multidrug resistant *Shigella sonnei* isolates from an outbreak in Thimphu, Bhutan. BMC Res Notes 7: 95–104. |
| Bolivia | Townes JM, Quick R, Gonzales OY, Linares M, Damiani E, et al. (1997) Etiology of Bloody Diarrhea in Bolivian Children: Implications for Empiric Therapy. J Infect Dis 175: 1527–1530. |
| Botswana | Rowe JS, Shah SS, Motlhagodi S, Bafana M, Tawanana E, et al. (2010) An epidemiologic review of enteropathogens in Gaborone, Botswana: shifting patterns of resistance in an HIV endemic region. PLoS One 5: e10924. |
| Brazil | Da Cruz CBN, de Souza MCS, Serra PT, Santos I, Balieiro A, et al. (2014) Virulence factors associated with pediatric shigellosis in Brazilian Amazon. Biomed Res Int 2014: 539697. |
| Bulgaria | Bratoeva MP, John JF, Barg NL (1992) Molecular Epidemiology of Trimethoprim-Resistant *Shigella boydii* Serotype 2 Strains from Bulgaria. J Clin Microbiol 30: 1428–1431. |
| Burkina Faso | Bonkoungou IJO, Haukka K, Österblad M, Hakanen AJ, Traoré AS, et al. (2013) Bacterial and viral etiology of childhood diarrhea in Ouagadougou, Burkina Faso. BMC Pediatr 13: 36–42. |
| Cambodia | Study: Meng CY, Smith BL, Bodhidatta L, Richard SA, Vansith K, et al. (2011) Etiology of Diarrhea in Young Children and Patterns of Antibiotic Resistance in Cambodia. Pediatr Infect Dis J 30: 331–335.  Species data: Dr Ladaporn Bodhidatta, Armed Forces Research Institute of Medical Sciences, Bangkok, Thailand, Department of Enteric Diseases. |
| Canada | Canadian Integrated Surveillance Report: Salmonella, Campylobacter, verotoxigenic E. coli and *Shigella*, from 2000-2004 (2009). Available at http://www.phac-aspc.gc.ca/publicat/ccdr-rmtc/09vol35/35s3/index-eng.php |
| Central African Republic | Bercion R, Njuimo SP, Boudjeka PM, Manirakiza A (2008) Distribution and antibiotic susceptibility of *Shigella* isolates in Bangui, Central African Republic. Trop Med Int Heal 13: 468–471. |
| Chile | Hamilton-West C, J VP, Carlos J, Hormazábal O, Z RL, et al. (2007) Epidemiología clínica y molecular de las infecciones por *Shigella* spp en ninos de la Region Metropolitana durante el verano 2004-2005. Rev Med Chil 135: 1388–1396. |
| China | Zhang J, Wang F, Jin H, Hu J, Yuan Z, et al. (2014) Laboratory monitoring of bacterial gastroenteric pathogens *Salmonella* and *Shigella* in Shanghai, China 2006-2012. Epidemiol Infect: 1–8. |
| Colombia | Urbina D, Arzuza O, Young G, Parra E, Castro R, et al. (2003) Rotavirus type A and other enteric pathogens in stool samples from children with acute diarrhea on the Colombian northern coast. Int Microbiol 6: 27–32. |
| Costa Rica | Achi R, Mata L, Siles X, Lindberg A (1996) Immunomagnetic Separation and PCR Detection Show *Shigellae* to be Common Faecal Agents in Children From Urban Marginal Communities of Costa Rica. J Infect 32: 211–218. |
| Côte d'Ivoire | Antoine B, Adjehi D, Nathalie G, Valerie G, Etienne D, et al. (2010) Virulence Factors and Resistance Profile of *Shigella* Isolated During Infectious Diarrhea in Abidjan, Côte D’ Ivoire. J Appl Sci Res 6: 594–599. |
| Cuba | Bravo LF, Correa Y, Clausell JF, Fernandez A, Ramírez M, et al. (2009) Caracterización de factores de virulencia y susceptibilidad antimicrobiana en cepas de *Plesiomonas shigelloides* aisladas de pacientes con diarrea aguda en Cuba. Microbiol Clin 26: 233–238. |
| Denmark | Berger S (2014) Shigellosis: Global Status - 2014 edition. Gideon Informatics. |
| Djibouti | Mikhail IA, Fox E, Haberberger L, Ahmed MH, Abbatte EA (1990) Epidemiology of Bacterial Pathogens Associated with Infectious Diarrhea in Djibouti. J Clin Microbiol 28: 956–961. |
| Ecuador | Sempertegui F, Estrella B, Egas J, Carrion P, Yerovi L, et al. (1995) Risk of diarrheal disease in Ecuadorian day-care centers. Pediatr Infect Dis J 14: 606–612. |
| Egypt | Ahmed S, Riddle M, Wierzba R, Messih IA, Monteville M, et al. (2006) Epidemiology and genetic characterization of *Shigella flexneri* strains isolated from three paediatric populations in Egypt (2000-2004). Epidemiol Infect 134: 1237–1248. |
| Eritrea | Naik D (2006) Prevalence and antimicrobial susceptibility patterns of *Shigella* species in Asmara, Eritrea, northeast Africa. J Microbiol Immunol Infect 39: 392–395. |
| Ethiopia | Asrat D (2008) *Shigella* and *Salmonella* serogroups and their antibiotic susceptibility patterns in Ethiopia. Le Rev Sante la Mediterr Orient 14: 760–767. |
| Fiji | Watson C (2006) Death from Multi-resistant shigelloses: a case study from Fiji. Pacific Public Heal 13: 111–114. |
| Finland | Haukka K, Siitonen A (2008) Emerging resistance to newer antimicrobial agents among *Shigella* isolated from Finnish foreign travellers. Epidemiol Infect 136: 476–482. |
| France | Berger S (2014) Shigellosis: Global Status - 2014 edition. Gideon Informatics. |
| Gabon | Schaumburg F, Alabi AS, Kaba H, Lell B, Becker K, et al. (2014) Molecular characterization of *Shigella* spp. from patients in Gabon 2011 – 2013. Trans R Soc Trop Med Hyg Nov 20: pii:tru175. |
| Germany | Robert Koch Institute: Shigellosis (2013). Robert Koch Inst SurvStat@RKI. Available at http://www3.rki.de/SurvStat/QueryForm.aspx. |
| Ghana | Opintan JA, Newman MJ, Ayeh-Kumi PF, Affrim R, Gepi-Attee R, et al. (2010) Pediatric diarrhea in southern Ghana: etiology and association with intestinal inflammation and malnutrition. Am J Trop Med Hyg 83: 936–943. |
| Guatemala | Cruz JR, Cano F, Bartlett A V, Mendez H (1994) Infection, diarrhea, and dysentery caused by *Shigella* species and Campylobacter jejuni among Guatemalan rural children. Pediatr Infect Dis J 13: 216–223. |
| Hungary | Nogrady N, Kiraly M, Borbas K, Toth A, Paszti J, et al. (2013) Antimicrobial resistance and genetic characteristics of integron-carrier shigellae isolated in Hungary. J Med Microbiol 62: 1545–1551. |
| India | Livio S, Strockbine NA, Panchalingam S, Tennant SM, Barry EM, et al. (2014) *Shigella* isolates from the global enteric multicenter study inform vaccine development. Clin Infect Dis 59: 933–941. |
| Indonesia | Herwana E, Surjawidjaja JE, Salim OC, Indriani N, Bukitwetan P, et al. (2010) *Shigella*-associated diarrhea in children in South Jakarta, Indonesia. Southeast Asian J Trop Med Public Health 41: 418–425. |
| Iran | Jomezadeh N, Babamoradi S, Kalantar E, Javaherizadeh H (2014) Isolation and antibiotic susceptibility of *Shigella* species from stool samples among hospitalized children in Abadan, Iran. Gastroenterol Hepatol from Bed to Bench 7: 218–223. |
| Iraq | Mohammed AMN (2009) Shigellae – associated diarrhoea in children in Baghdad – Iraq. Iraqi J Med Sci 7: 59–65. |
| Ireland | Annual Report 2012: Health Protection Surveillance Centre (2012). Dublin. Available at http://www.hpsc.ie/AboutHPSC/AnnualReports/File,14421,en.pdf |
| Israel | Cohen D, Bassal R, Goren S, Rouach T, Taran D, et al. (2014) Recent trends in the epidemiology of shigellosis in Israel. Epidemiol Infect 142: 2583–2594. |
| Japan | Infectious Disease Surveillance Center (IDSC) of Japan: *Shigella* (2012). Available at http://idsc.nih.go.jp/. |
| Jordan | Gargouri N, Walke H, Belbeisi A, Hadadin A, Salah S, et al. (2009) Estimated Burden of Human *Salmonella, Shigella*, and Brucella Infections in Jordan, 2003–2004. Foodborne Pathog Dis 6: 481–487. |
| Kenya | Livio S, Strockbine NA, Panchalingam S, Tennant SM, Barry EM, et al. (2014) *Shigella* isolates from the global enteric multicenter study inform vaccine development. Clin Infect Dis 59: 933–941. |
| Kuwait | Jamal W, Rotimi VO, Pal T, Sonnevend A, Dimitrov TS (2010) Comparative in vitro activity of tigecycline and other antimicrobial agents against *Shigella* species from Kuwait and the United Arab of Emirates. J Infect Public Health 3: 35–42. |
| Laos | Phetsouvanh R, Midorikawa Y, Nakamura S (1999) The seasonal variation in the microbial agents implicated in the etiology of diarrheal diseases among children in Lao People’s Democratic Republic. Southeast Asian J Trop Med Public Health 30: 319–324. |
| Lebanon | Araj GF, Avedissian AZ, Ayyash NS, Bey HA, Asmar RG El, et al. (2012) A reflection on bacterial resistance to antimicrobial agents at a major tertiary care center in Lebanon over a decade. Leban Med J 60: 125–135. |
| Liberia | Guyot A (1996) Antibiotic resistance in *Shigella* in Monrovia. Trop Doct 26: 70–71. |
| Libya | Ali MB, Ghenghesh KS, Aissa RB, Abuhelfaia A, Dufani M (2005) Etiology of childhood diarrhea in Zliten, Libya. Saudi Med J 26: 1759–1765. |
| Lithuania | Jensen G, Wandall D, Gaarslev K, Panavas S, Gutschik E (1996) Antibiotic Resistance in *Shigella* and *Salmonella* in a Region of Lithuania. Eur J Clin Microbiol Infect Dis 15: 872–876. |
| Madagascar | Randrianirina F, Ratsima EH, Ramparany L, Randremanana R, Rakotonirina HC, et al. (2014) Antimicrobial resistance of bacterial enteropathogens isolated from stools in Madagascar. BMC Infect Dis 14: 104. |
| Malawi | Pitman C, Amali R, Kanyerere H, Siyasiya A, Phiri S, et al. (1996) Bloody diarrhoea of adults in Malawi: antimicrobial sensitivities clinical features, infectious agents, and antimicrobial sensitivities. Trans R Soc Trop Med Hyg 90: 284–287. |
| Malaysia | Banga Singh K-K, Ojha SC, Deris ZZ, Rahman RA (2011) A 9-year study of shigellosis in Northeast Malaysia: Antimicrobial susceptibility and shifting species dominance. Zeitschrift fur Gesundheitswissenschaften 19: 231–236. |
| Mali | Livio S, Strockbine NA, Panchalingam S, Tennant SM, Barry EM, et al. (2014) *Shigella* isolates from the global enteric multicenter study inform vaccine development. Clin Infect Dis 59: 933–941. |
| Mexico | Zaidi MB, Estrada-García T, Campos FD, Chim R, Arjona F, et al. (2013) Incidence, clinical presentation, and antimicrobial resistance trends in *Salmonella* and *Shigella* infections from children in Yucatan, Mexico. Front Microbiol 4: 288. |
| Montenegro | Sipetic-Grujicic S, Glusac S, Ratkov I, Maksimovic J, Ratkov E, et al. (2010) Shigellosis - epidemiological situation in Montenegro in period 1996-2005 [Serbian]. Med Pregl 63: 554–557. |
| Mozambique | Livio S, Strockbine NA, Panchalingam S, Tennant SM, Barry EM, et al. (2014) *Shigella* isolates from the global enteric multicenter study inform vaccine development. Clin Infect Dis 59: 933–941. |
| Myanmar | Oo K, M T (1995) Serotype distribution and antimicrobial susceptibility of *Shigellae* isolated from diarrhoeal patients in Yangon, Myanmar. J Diarrhoeal Dis Res 13: 180–182. |
| Nepal | Kansakar P, Baral P, Malla S, Ghimire GR (2004) Antimicrobial susceptibilities of enteric bacterial pathogens isolated in Kathmandu, Nepal, during 2002-2004. J Infect Dev Ctries 5: 163–168. |
| Netherlands | Van Pelt W, de Wit M, Wannet W, Ligtvoet E, Widdowson M, et al. (2003) Laboratory surveillance of bacterial gastroenteric pathogens in The Netherlands, 1991 – 2001. Epidemiol Infect 130: 431–441. |
| Nigeria | Abdu A, Aboderin AO, Elusiyan JB, Kolawole D, Lamikanra A (2013) Serogroup distribution of *Shigella* in Ile-Ife, southwest Nigeria. Trop Gastroenterol 34: 164–169. |
| Oman | Patel P, Mercy J, Shenoy J, Ashwini B (2008) Factors associated with acute diarrhoea in children in Dhahira, Oman: a hospital-based study. East Mediterr Heal J 14: 571–578. |
| Pakistan | Livio S, Strockbine NA, Panchalingam S, Tennant SM, Barry EM, et al. (2014) *Shigella* isolates from the global enteric multicenter study inform vaccine development. Clin Infect Dis 59: 933–941. |
| Papua New Guinea | Greenhill AR, Guwada C, Siba V, Michael A, Yoannes M, et al. (2014) Antibiotic resistant *Shigella* is a major cause of diarrhoea in the Highlands of Papua New Guinea. J Infect Dev Ctries 8: 1391–1397. |
| Paraguay | Basualdo W, Arbo A (2003) Randomized comparison of azithromycin versus cefixime for treatment of shigellosis in children. Pediatr Infect Dis J 22: 374–377. |
| Peru | Kosek M, Yori PP, Pan WK, Olortegui MP, Gilman RH, et al. (2013) Epidemiology of Highly Endemic Multiply Antibiotic-Resistant Shigellosis in Children in the Peruvian Amazon. Pediatrics 122: e541–549. |
| Poland | Stypulkowska-Misiurewicz H, Baumann-Popczyk A (2013) Shigellosis in Poland in 2011. Przegl Epidemiol 67: 217–219. |
| Romania | Luca C, Nemescu R, Teodor A, Fantanaru R, Petrovici C, et al. (2011) Etiological aspects of acute gastroenteritis - a ten year review. Rev Med Chir Soc Med Nat Iasi 115: 712–717. |
| Russia | Berger S (2014) Shigellosis: Global Status - 2014 edition. Gideon Informatics. |
| Rwanda | Bogaerts J, Verhaegen J, Munyabikali JP, Mukantabana B, Lemmens P, et al. (1997) Antimicrobial Resistance and Serotypes of *Shigella* Isolates in Kigali, Rwanda (1983 to 1993): Increasing Frequency of Multiple Resistance. Bacteriology 28: 165–171. |
| Saudi Arabia | Panhotra BR, Saxena AK, Al-Mulhim K (2004) Emergence of Nalidixic Acid Resistance in *Shigella sonnei* Isolated from Patients Having Acute Diarrheal Disease: Report from Eastern Province of Saudi Arabia. Japanese J Infecious Dis 57: 116–118. |
| Senegal | Sire J-M, Garin B, Chartier L, Fall NK, Tall A, et al. (2013) Community-acquired infectious diarrhoea in children under 5 years of age in Dakar, Senegal. Paediatr Int Child Health 33: 139–144. |
| Serbia | Berger S (2014) Shigellosis: Global Status - 2014 edition. Gideon Informatics. |
| Slovakia | Berger S (2014) Shigellosis: Global Status - 2014 edition. Gideon Informatics. |
| Somalia | Casalino M, Nicoletti M, Salvia A, Colonna B, Pazzani C, et al. (1994) Characterization of Endemic *Shigella flexneri* Strains in Somalia: Antimicrobial Resistance, Plasmid Profiles, and Serotype Correlation. J Clin Microbiol 32: 1179–1183. |
| South Africa | Tau NP, Smith AM, Sooka A, Keddy KH (2009) Molecular characterization of extended-spectrum beta-lactamase-producing *Shigella* isolates from humans in South Africa, 2003-2009. J Med Microbiol 61: 162–164. |
| South Korea | Lee JC, Jeong YS, Oh JY, Kang HY, Kim KH, et al. (2006) Epidemiology of Shigellosis in Korea. J Bacteriol Virol 36: 41–49. |
| Spain | Hernández AC, Calvo AV, Lobato ES, Jiménez FF (2008) Infección por *Shigella* spp. en el Hospital de Getafe entre 2001 y 2006. An Pediatr 68: 605–609. |
| Sudan | Ahmed AA, Osman H, Mansour AM, Musa HA, Ahmed AB, et al. (2000) Antimicrobial agent resistance in bacterial isolates from patients with diarrhea and urinary tract infection in the Sudan. Am J Trop Med Hyg 63: 259–263. |
| Sweden | Svenungsson B, Lagergren A, Ekwall E, Evengard B, Hedlund KO, et al. (2000) Enteropathogens in Adult Patients with Diarrhea and Healthy Control Subjects: A 1-Year Prospective Study in a Swedish Clinic for Infectious Diseases. Clin Infect Dis 30: 770–778. |
| Switzerland | Berger S (2014) Shigellosis: Global Status - 2014 edition. Gideon Informatics. |
| Taiwan | Wu C-H, Huang L-T, Huang I-F, Liu J-W, Chen J-B, et al. (2009) Acute non-outbreak shigellosis: ten years experience in southern Taiwan. Chang Gung Med J 32: 59–65. |
| Tanzania | Moyo SJ, Gro N, Matee MI, Kitundu J, Myrmel H, et al. (2011) Age specific aetiological agents of diarrhoea in hospitalized children aged less than five years in Dar es Salaam, Tanzania. BMC Pediatr 11: 19–25. |
| Thailand | Bangtrakulnonth A, Vieira AR, Lo Fo Wong DM a, Pornreongwong S, Pulsrikarn C, et al. (2008) *Shigella* from humans in Thailand during 1993 to 2006: spatial-time trends in species and serotype distribution. Foodborne Pathog Dis 5: 773–784. |
| The Gambia | Livio S, Strockbine NA, Panchalingam S, Tennant SM, Barry EM, et al. (2014) *Shigella* isolates from the global enteric multicenter study inform vaccine development. Clin Infect Dis 59: 933–941. |
| Trinidad & Tobago | Orrett FA (2008) Prevalence of *Shigella* Serogroups and Their Antimicrobial Resistance Patterns in Southern Trinidad. J Health Popul Nutr 26: 456–462. |
| Tunisia | Al-Gallas N, Bahri O, Bouratbeen A, Haasen A Ben, Aissa R Ben (2007) Etiology of Acute Diarrhea in Children and Adults in Tunis, Tunisia, with Emphasis on Diarrheagenic Escherichia coli: Prevalence, Phenotyping, and Molecular Epidemiology. Am J Trop Med Hyg 77: 571–582. |
| Turkey | Saran B, Erdem B, Tekelİ FA, Şahİn F, Aysev AD (2013) Ankara’ da İzole Edilen *Shigella* Kökenlerinin Antibiyotik Direnç Modelleri, Plazmid Profil Analizi ve Değişken Alanlı Jel Elektroforezi ile İncelenmesi (Characterization of *Shigella* Strains Isolated in Ankara, Turkey by Antimicrobial Resistance Models, Pl. Mikrobiyol Bul 47: 35–48. |
| United Arab Emirates | Jamal W, Rotimi VO, Pal T, Sonnevend A, Dimitrov TS (2010) Comparative in vitro activity of tigecycline and other antimicrobial agents against *Shigella* species from Kuwait and the United Arab of Emirates. J Infect Public Health 3: 35–42. |
| United Kingdom | Shigella cases: 1992 to 2013 (2013). Public Heal Engl Stat. Available: https://www.gov.uk/government/statistics/shigella-cases-1992-to-2013. |
| United States | National Antimicrobial Resistance Monitoring System: Enteric Bacteria (2011). Atlanta, Georgia. Available at http://www.cdc.gov/narms/pdf/2011-annual-report-narms-508c.pdf |
| Uruguay | Mota M, Gadea M, González S, González G, Pardo L, et al. (2010) Bacterial pathogens associated with bloody diarrhea in Uruguayan children. Rev Argent Microbiol 42: 114–117. |
| Uzbekistan | Madiyarov RS, Bektemirov AM, Ibadova G a, Abdukhalilova GK, Khodiev A V, et al. (2010) Antimicrobial resistance patterns and prevalence of class 1 and 2 integrons in *Shigella flexneri* and *Shigella sonnei* isolated in Uzbekistan. Gut Pathog 2: 18–24. |
| Venezuela | Pérez-Schael I, Salinas B, González R, Salas H, Ludert JE, et al. (2007) Rotavirus mortality confirmed by etiologic identification in Venezuelan children with diarrhea. Pediatr Infect Dis J 26: 393–397. |
| Vietnam | Vinh H, Nhu NTK, Nga TVT, Duy PT, Campbell JI, et al. (2009) A changing picture of shigellosis in southern Vietnam: shifting species dominance, antimicrobial susceptibility and clinical presentation. BMC Infect Dis 9. |
| Yemen | Al-Moyed K, Harmal N, Al-Harasy A, Al-Shamahy H (2006) Increasing single and multi-antibiotic resistance in *Shigella* species isolated from shigellosis patients in Sana’a, Yemen. Saudi Med J 27: 1157–1160. |
| Zimbabwe | Ndlovu N, Tarupiwa A, Mudzori J (2006) Antimicrobial resistance of *Shigella* species isolated during 2004 and 2005 from selected sites in Zimbabwe. Cent Afr J Med 52: 93–97. |
