# Supplementary material for: The Rising Dominance of Shigella sonnei: An Intercontinental Shift in the Etiology of Bacillary Dysentery
Source: PLoS Negl Trop Dis. 2015 Jun 11;9(6):e0003708. doi: 10.1371/journal.pntd.0003708 (PMC4466244; doi:10.1371/journal.pntd.0003708)
Supplement: S1 Text — Data are derived from analyses of 367 Shigella isolates collected between 1995 and 2010 from Vietnam (136 S. flexneri and 231 S. sonnei). Resistance was determined by MIC and gene content analysis from Illumina genome sequencing data. (DOCX) [file pntd.0003708.s002.docx]

**Text S1. Methods for antimicrobial resistance and gene content data shown in Figure 2.** A total of 136 *Shigella flexneri* and 231 *Shigella sonnei* isolates were collected between 1995 and 2010 as part of ongoing studies across Vietnam [1,2]. Antimicrobial susceptibilities were tested at the time of isolation by the modified Bauer-Kirby disk diffusion method, as recommended by the CLSI guidelines [3]. MICs were accessed by E-test, according to the manufacturer’s recommendations (AB Biodisk). Mueller-Hinton agar and antimicrobial discs were purchased from Unipath, Basingstoke, United Kingdom. Escherichia coli ATCC 25922 was used as the control strain. The inhibitory zone sizes were recorded and interpreted according to current CLSI breakpoint guidelines [3]. The following antimicrobials were used for *Shigella.spp* susceptibility testing: ampicillin (AMP), chloramphenicol (CHL), ciprofloxacin (CIP), ceftriaxone (CRO), gatifloxacin (GAT), gentamicin (GEN), nalidixic acid (NAL), ofloxacin (OFX), trimethoprim/ sulfamethoxazole (SXT), tetracycline (TET) and trimethoprim (TMP).

Additionally, the isolates were subjected to DNA extraction by using Wizard Genomic DNA Extraction Kit (Promega, Wisconsin, USA), and whole genome sequenced on an Illumina Hiseq2000 platform (Illumina, San Diego USA) to generate 150bp paired-end reads, as previously described [4]. *De novo* assemblies were generated for each read set using Velvet and VelvetOptimiser [5]. Contigs less than 100bp in size were excluded for further analysis. The short-read sequence data were deposited in the European Read Archive under the accession number ERP000182, ERP000631 and ERP000631. The resistance gene profiles and content (resistome) of each isolate were characterised using a manually curated database, based on the ResFinder database [6]. Each gene in the database was mapped against the isolate assemblies to identify complete genes. Where fragments matching the 5´or 3´ ends of resistance genes were identified at contig boundaries, sequencing reads were mapped to all matching candidate genes and their presence assessed based on mapping coverage across the gene.

1. Holt K, Thieu Nga T, Thanh D, Vinh H, Kim D, et al. (2013) Tracking the establishment of local endemic populations of an emergent enteric pathogen. Proc Natl Acad Sci 110: 17522–17527.

2. Holt KE, Baker S, Weill F-X, Holmes EC, Kitchen A, et al. (2012) Shigella sonnei genome sequencing and phylogenetic analysis indicate recent global dissemination from Europe. Nat Genet 44: 1056–1059.

3. Clinical and Laboratory Standards Institute (2012) Performance standards for antimicrobials disk susceptibility test. Wayne, PA.

4. Quail MA, Kozarewa I, Smith F, Scally A, Stephens PJ, et al. (2008) A large genome center ’ s improvements to the Illumina sequencing system. Nat Methods 5: 1005–1010.

5. Zerbino DR, Birney E (2008) Velvet: algorithms for de novo short read assembly using de Bruijn graphs. Genome Res 18: 821–829.

6. Zankari E, Hasman H, Cosentino S, Vestergaard M, Rasmussen S, et al. (2012) Identification of acquired antimicrobial resistance genes. J Antimicrob Chemother 67: 2640–2644.
